# Supplementary material for: A Roman provincial city and its contamination legacy from artisanal and daily-life activities
Source: PLoS One. 2021 Jun 9;16(6):e0251923. doi: 10.1371/journal.pone.0251923 (PMC8189455; doi:10.1371/journal.pone.0251923)
Supplement: S1 Table — (PDF) [file pone.0251923.s002.pdf]

Test	Element	F	num df	denom df	p-value
A	Pb	13.114	3	23.3	3.19E-05
B	Pb	16.904	2	23.523	2.82E-05
A	Cu	38.946	3	46.5	9.71E-13
B	Cu	52.693	2	35.249	2.56E-11
A	Al	62.846	3	20.312	1.87E-10
B	Al	15.82	2	12.408	0.0003858
A	Zn	38.092	3	16.595	1.16E-07
B	Zn	15.831	2	9.8403	0.0008406
A	Zr	33.699	3	16.818	2.46E-07
B	Zr	0.25659	2	10.007	0.7786
A	Ca	16.103	3	16.811	3.41E-05
B	Ca	0.7675	2	10.018	0.4896
A	Ti	42.954	3	17.037	3.69E-08
B	Ti	0.82016	2	10.169	0.4675
B	K	76.667	2	17.729	1.88E-09
B	Ag	53.66	2	35.685	1.75E-11
B	Sn	27.211	2	24.152	6.50E-07
B	As	7.1228	2	13.832	0.007473
