# Supplementary material for: A Roman provincial city and its contamination legacy from artisanal and daily-life activities
Source: PLoS One. 2021 Jun 9;16(6):e0251923. doi: 10.1371/journal.pone.0251923 (PMC8189455; doi:10.1371/journal.pone.0251923)
Supplement: S2 Table — (PDF) [file pone.0251923.s003.pdf]

Element	Location	diff	ci.lo	ci.hi	t	df	p
Pb	Off-Bed	11.54	5.558	17.52	5.037	14.32	0.0005
Pb	On-Bed	121.04	26.794	215.28	3.216	23.08	0.0103
Pb	On-Off	109.5	15.257	203.74	2.909	23.09	0.0207
Ca	Off-Bed	2.308	-29.682	34.298	0.2541	4.105	0.9653
Ca	On-Bed	3.898	-28.163	35.959	0.4303	4.061	0.9052
Ca	On-Off	1.59	-1.536	4.716	1.2258	54.379	0.4433
Ti	Off-Bed	0.06151	-0.18826	0.31128	0.8518	4.278	0.6934
Ti	On-Bed	0.08185	-0.16565	0.32935	1.1175	4.526	0.549
Ti	On-Off	0.02034	-0.03361	0.07429	0.9139	44.903	0.6344
Al	Off-Bed	1.2274	0.3577	2.097	4.331	5.996	0.0117
Al	On-Bed	1.8163	0.9335	2.699	5.772	8.769	0.0008
Al	On-Off	0.5889	0.0536	1.124	2.673	41.999	0.0282
Cu	Off-Bed	7.039	4.095	9.983	5.874	32.18	0.0001
Cu	On-Bed	19.65	14.336	24.964	9.194	25.72	0.0001
Cu	On-Off	12.611	6.937	18.284	5.44	34.98	0.0001
Zn	Off-Bed	-5.701	-77.67	66.27	0.2804	4.059	0.958
Zn	On-Bed	12.16	-59.58	83.9	0.5957	4.126	0.8297
Zn	On-Off	17.861	10.4	25.32	5.8144	42.894	0.0001
Zr	Off-Bed	12.121	-60.86	85.11	0.581	4.167	0.8368
Zr	On-Bed	14.218	-58.25	86.69	0.6749	4.335	0.8368
Zr	On-Off	2.098	-10.35	14.55	0.4087	43.915	0.8368
K	Off-Bed	0.3678	0.2438	0.4919	8.05	10.63	0.0001
K	On-Bed	0.716	0.569	0.863	12.357	19.37	0.0001
K	On-Off	0.3482	0.216	0.4804	6.407	40.38	0.0001
Ag	Off-Bed	113.7	81.42	145.9	8.662	32	0.0001
Ag	On-Bed	319.9	182.85	457	5.845	23.00	0.0001
Ag	On-Off	206.2	66.28	346.2	3.664	25.66	0.0031
Sn	Off-Bed	0.9333	0.4086	1.458	4.584	16.21	0.0008
Sn	On-Bed	2.5042	1.6026	3.406	6.887	27	0.0001
Sn	On-Off	1.5708	0.6659	2.476	4.265	32.12	0.0005
As	Off-Bed	2.421	0.05207	4.789	2.982	7.274	0.0457
As	On-Bed	3.468	1.00169	5.935	3.811	10.744	0.0078
As	On-Off	1.048	-0.69747	2.793	1.456	44.084	0.3217
