# Supplementary material for: A Roman provincial city and its contamination legacy from artisanal and daily-life activities
Source: PLoS One. 2021 Jun 9;16(6):e0251923. doi: 10.1371/journal.pone.0251923 (PMC8189455; doi:10.1371/journal.pone.0251923)
Supplement: S1 File — (DOCX) [file pone.0251923.s004.docx]

A Roman provincial city and its contamination legacy from artisanal and daily-life activities

Genevieve Holdridge, Søren M Kristiansen, Gry H Barfod, Tim C. Kinnaird, Achim Lichtenberger, Jesper Olsen, Bente Philippsen, Rubina Raja, Ian Simpson

Corresponding author: Søren M. Kristiansen

Email: smk@geo.au.dk

**This PDF file includes:**

Supplementary text

Figures S1 to S9

Legends for Datasets S1

Legends for Table S1 to S2.

SI References

**Supplementary Information Text**

**Principal Component Analysis**

Data were analyzed statistically using descriptive statistics and Principal Components Analysis (PCA) according to guidelines by Esbensen et al (1). The PCA was applied to the geochemical results from the ICPMS dataset to explore the overall data structure. The dataset consists of 36 elements (variables) for 62 samples (objects). The PCA was based on a correlations matrix, and dataset first was standardized and normalized by subtracting mean values of each element from the value of the element and then taking 1/STD of the element multiplied with √(N_1). All PCA calculations and figures were made in Python 3.6 (the Numpy, Pandas, scikit-learn and Matplotlib modules).

**Enrichment Factors**

Enrichment factors were calculated on the values of several of the elements including Ti, Zr, Ca, Pb, Sn, As, Ag, and Cu for the sediment and soils in the wadi and from the Northwest Quarter of Jerash as compared to the average values of these elements in the local bedrock. The following calculation was used according to (2):

EF = (Vsed / Vref) / (VRsed / VRref)

Where ER is enrichment factor, Vsed is the value of the element of the sediment, and Vref is the value of the element in the bedrock. The calculated EF represents the normalized relationship between the bedrock value of an element and the value in the sediment and is calculated by dividing the ratio of value of the element under consideration in both the sediment and bedrock by the ratio of the value of a reference element in both the sediment and bedrock.

**Additional discussion of elemental distributions**

According to qualitative (comparative graph in Fig. S1) and quantitative analyses (e.g., PCA in Figs. S2, S3 and S4, and boxplots in Fig. S5) there was a clear contrast between the Pb and Cu heavy metal values from bedrock and on-site and offsite locations, as well as a clear difference between on-site and off-site Pb and Cu values (Table S1, S2. Furthermore, seventeen heavy metal values of Cu, Pb, Ag and Sn of 62 analyzed concentrations were found within the ancient city as compared to off-site locations (Dataset S1).

**
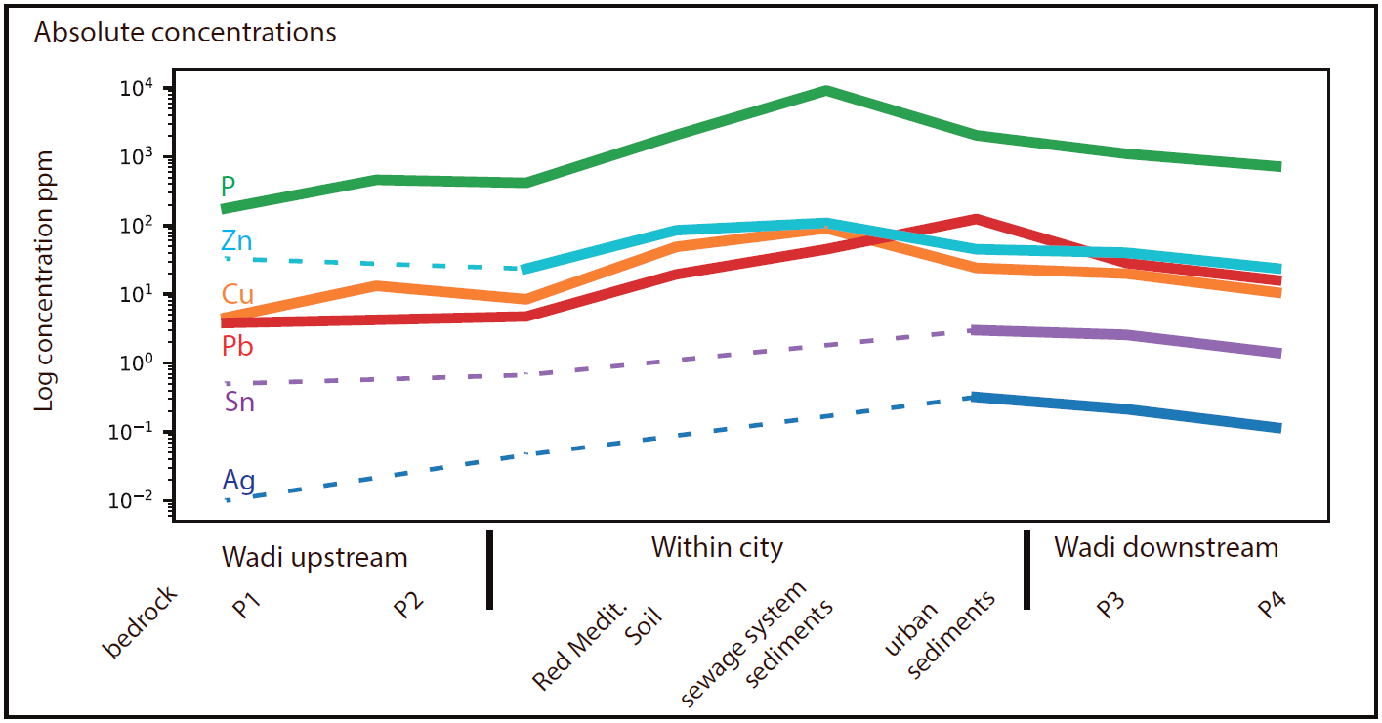
**

**Fig. S1.** Variations of concentrations of Cu, Pb, Zn, Ag, P and Sn found in soils and sediments upstream, within, and downstream of the ancient city of Jerash.

Several PCA models (Figs. S2, S3, S4) were tested and the model presented here was found to be very representative of these: PC1 was dominated by major elements and geology, whereas a group of anthropogenic elements (3) dominated PC2. Higher PCs were very variable and seemingly added little explanatory power. The PCA analysis also suggested that there is a clear distinction of elemental composition between sediments from ancient Jerash and the wadi (Fig. S4) as seen best on PC1 vs. PC2. Several clusters of elements appear to be important in affecting the spread of the data. One cluster includes several heavy metals, in particular, Pb, Cu, P, Sn, Ag, all clustered in the top part of Fig. S2 and Fig. S4b, which are considered typical anthropogenic indicators (3) and were more strongly linked to on-site profile sediments and soils. The other clusters of elements were related to bedrock. One cluster in the lower left of Fig. S2 involve Th, Ti, and Zr, which are common in sandstones, while Mg, Sr, Ca are located further to the right of the PCA loading plot and are usually in limestones, especially Ca. Sodium also appears to be an important influence, but whether Na can be related to the parent materials or to ancient drought is not clear as discussed in (4), but use of Na-rich irrigation water is a well-known agricultural issue in the area today (5).

**
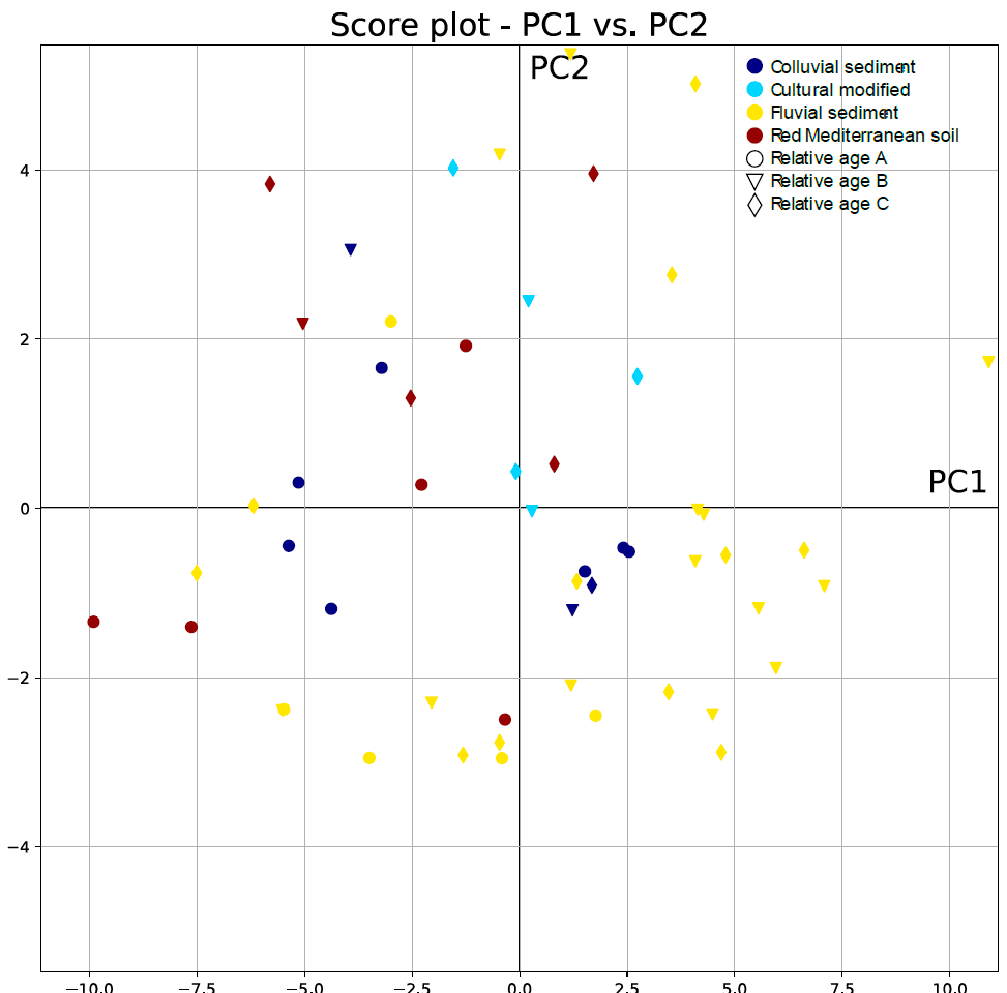
**

**Fig. S2.** Principle Component Analysis Score Plot indicating the relationship between different sediment/soil type (e.g., colluvial, fluvial, cultural, or Red Mediterranean Soil), and relative age (e.g., A is the earliest while C is the latest).

**
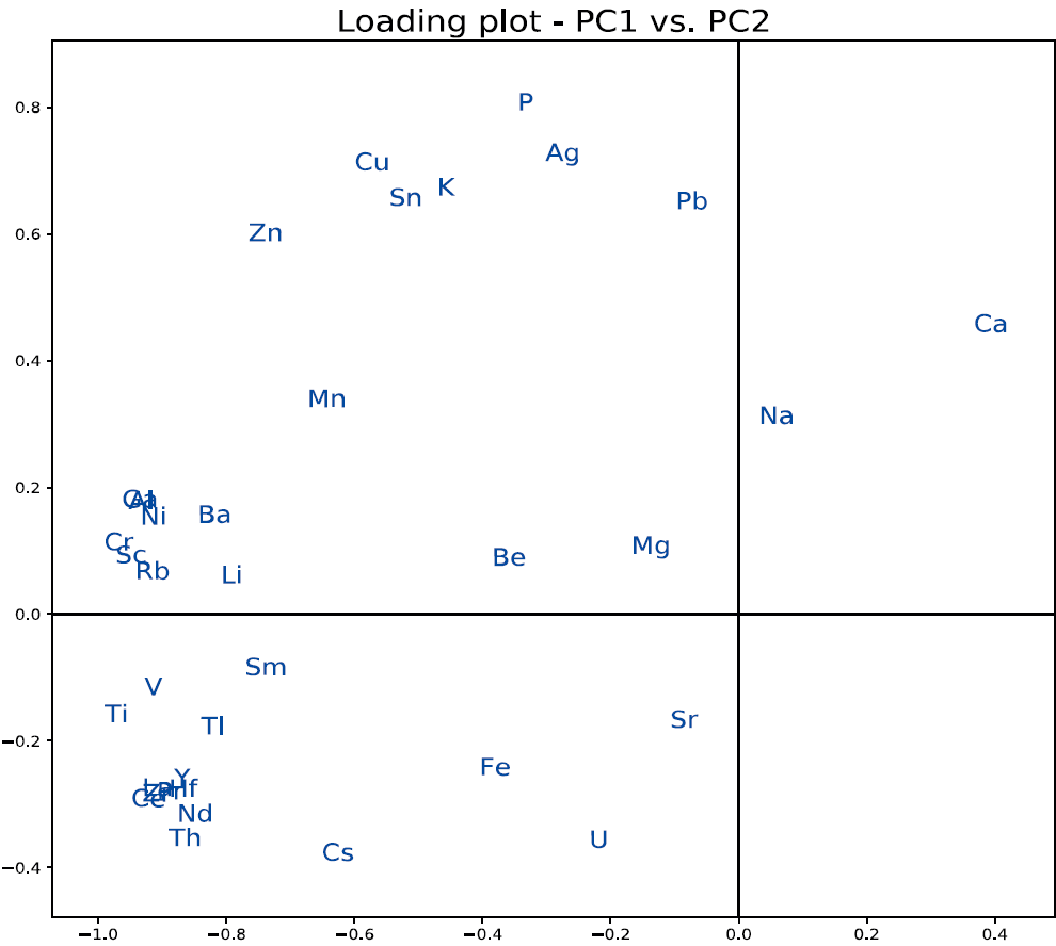
**

**Fig. S3.** Principle Component Analysis Loading Plot indicating the relationship between the elemental composition of different sediment/soil type (e.g., colluvial, fluvial, cultural, or Red Mediterranean Soil), and relative age (e.g., A is the earliest while C is the latest).

**
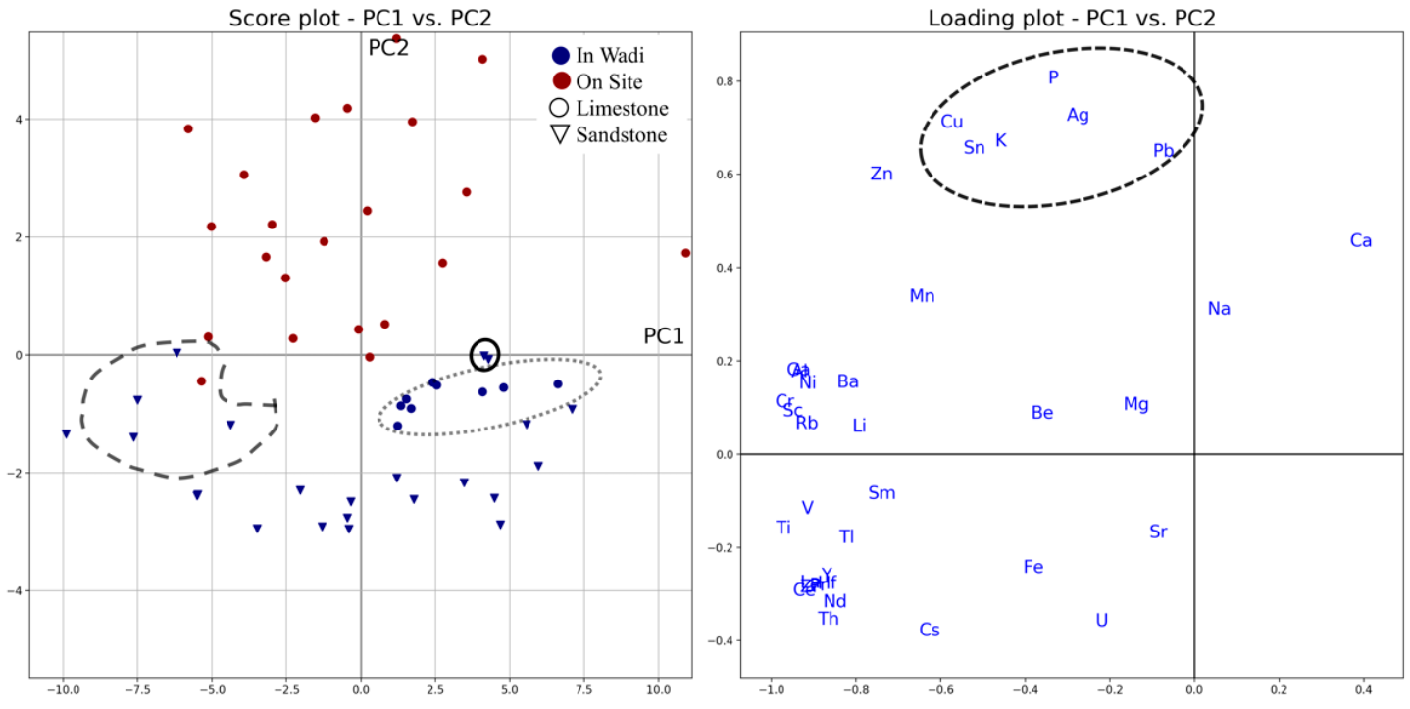
**

**Fig. S4.** Principle Component Analysis Score (a) and Loading Plots (b) indicating the relationship between bedrock type and on- and off-site locations. (a) The thick dashed circle on the left of the plot and the small solid black circle towards the right of the plot encompasses Profile P2 (middle section of Wadi Suf). The small dotted dashed circle to the right of the plot encompasses Profile P1 (upstream section of Wadi Suf). (b) The thick dashed circle at the top of the Score Plot encompasses the elements representing typical anthropogenic indicators including P, Ag, Pb, Cu and Sn (not K).

Examining the spread of heavy metal values along the wadi profiles in Dataset S1, the highest levels of Pb (20-30 ppm), Cu (12-24 ppm), Sn (1.5-3.5 ppm), and Ag (~150-300 ppb) were observed in Profile 2 followed by relatively high levels in Profile 3 listed as follows: Pb (10-24 ppm), Cu (4-18 ppm), Sn (0.5-2.4 ppm), and Ag (60-180 ppb). Conversely, Profile 1 had relatively low levels of Pb (4.75-7 ppm), Cu (6-9 ppm), Sn (0.5-0.9 %), and Ag (0-50 ppb). The boxplots (Fig. S5b and d) of the Pb and Cu in the wadi sediments highlight this distinct pattern of low amounts of contaminants in the upper wadi (Profile 1), highest amounts in the middle wadi (Profile 2), and relatively high amounts in the lower wadi (Profile 3). This pattern contrasts the observations of the other elements such as Ca, Ti, and Zr (Dataset S1) and further supports that the source for heavy metals, in particular Pb, in the wadi is derived from past activities in ancient Jerash and was only incorporated into sediments downstream. As example, Fig. S5 show a major difference in the spread of Pb and Cu concentrations on-site versus which is caused by the on-site pollution that affects the anthropogenic within city sediments only, the BN and NT samples, and not the bedrock and the the domestic outdoor space soi lin this case. The difference in the spread of the Pb and Cu concentrations in the on-site versus the off-site sediments (notice different y-axes) is likely caused by the input of contaminated soil into the wadi downstream of the city (sample site P2) where after non-polluted hinterland sediments again is washed into the wadi and dilutes the urban contamination signal.

**
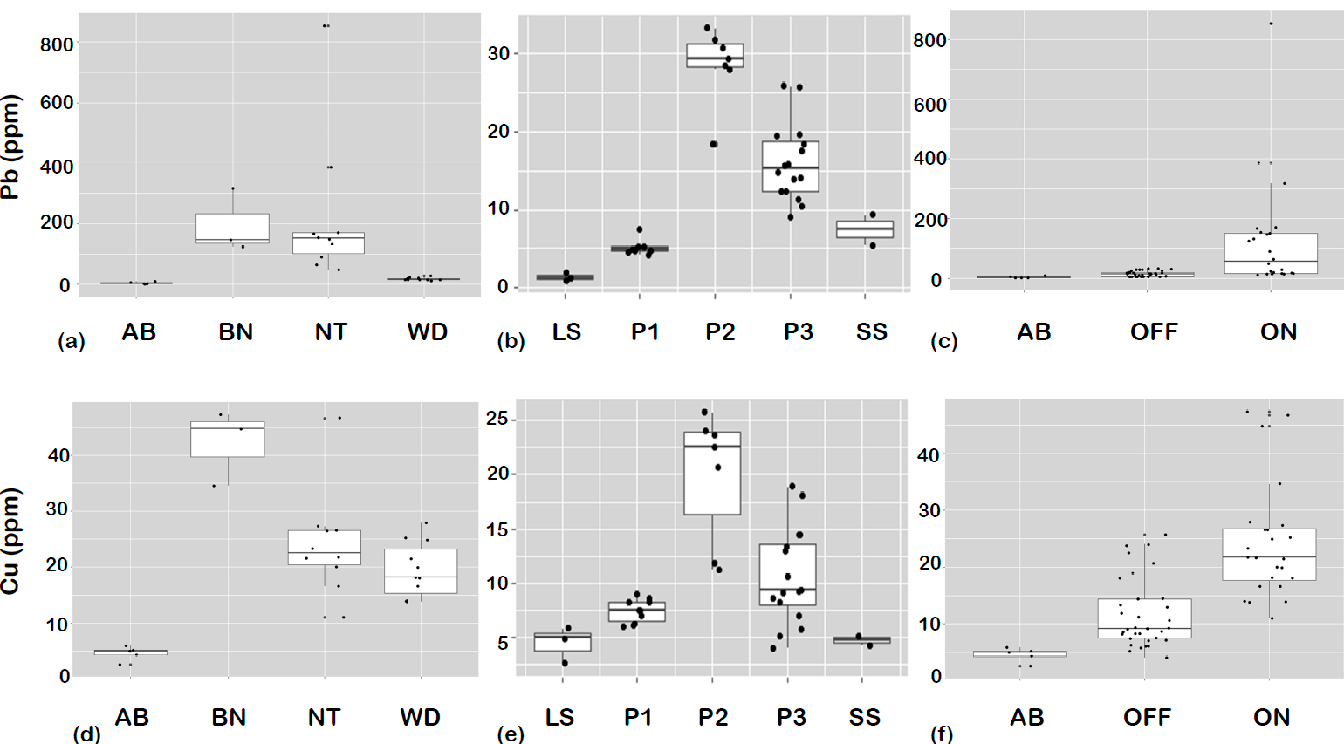
**

**Fig. S5.** Comparison between bedrock heavy metal values (combined sandstone and limestone = AB, all bedrock) and the three on-site profile (Profiles BN, NT and WD) sediment heavy metal values for Pb (a) and Cu (d). Comparison between bedrock heavy metal values (sandstone = SS and limestone = LS) and the three off-site profile (upper, middle and lower wadi, profiles 1, 2, and 3, respectively) sediment heavy metal values for Pb (b) and Cu (e). Comparison of combined bedrock heavy metal values (AB) against off-site (OFF) and on-site (ON) sediment heavy metal values for Pb (c) and Cu (f).

In addition, the heavy metal contamination and pollution observed in the soils and sediments along on-site stratigraphic profiles in the NWQ vary spatially and temporally. Fig. S1, the PCA analyses in Figs. S2, S3, S4, and the boxplots in Fig. S5 for on-site sediment heavy metal values indicate that in general, they were diverse in different spaces on site. For example, the overall lowest on-site values of Cu (14-25 ppm), Pb (12-22 ppm), and Sn (1-2 ppm) were located in the domestic outdoor space (e.g., Profile WD). In contrast, values of Cu (36-46 ppm), Pb (130-320 ppm), Ag (608-783), and Sn (5.4-6.4 ppm) were typically higher in outdoor spaces, such as the communal outdoor space represented by Profile BN, as well as along Profile NT, and in the sewage system sediments.

The past source for contaminants (e.g., legacy effects) can be discerned from modern surface pollutants (e.g., high levels of Pb and Ni and moderate levels of Cu and Cr) found in the Zarqa watershed because there is no discerned relationship with depth and no distinct connection to particle size along any of the profiles. The heavy metal values for on-site and off-site profiles vary with depth, so it appears there is no clear relationship with depth and in connection, age over time. Conversely, modern pollutants have a clear association with depth and particle size (6) Furthermore, absence of a relationship with depth suggests that these sediments were not altered during storage (i.e., by post-depositional transformation) or transport (7) and that fluctuations with depth reflects past source contributions (8-11). However, there are some trends such as along Profile BN, the Sn, Ag and Cu decrease from stratum [6] to stratum [4], whereas Pb increases between strata [6] to [4].

We also conducted statistical analyses on the heavy metal values using Welch’s ANOVA and post hoc Games Howell tests (userfriendlyscience package, R Software) due to unequal variances (Tables S1 and S2). Based on the results, statistical differences were observed between bedrock and sediment/soil values along on-site profiles (e.g., BN, NT, WD), and off-site profiles (e.g., P1, P2 and P3) for heavy metals Pb, Cu, Ag, As and Sn. Conversely, for reference heavy metals, Ti, Zr and Ca values were not statistically different from bedrock values, which also supports the use of Zr as a reference element in equation (8). Also, according to the statistical analyses comparing on- and off-site values, Pb and Ag were not statistically different between on-and off-site locations, but differences between the upper, middle and lower wadi profiles were significant at the 0.02 level. There was a significant difference between the three off-site stratigraphy profile values for Ag, Sn, As and Cu, at the 0.01 level, except for Cu, which had mixed results, including no significant difference between the upper and lower wadi profiles.

Enrichment factors (EFs) (Figs. S6, and S7) along the on-site profiles, in particular Profiles NT and BN, and to lesser extents along wadi Profiles P3 and P4 support that they are enriched in certain elements as compared to what is considered normal for local bedrock and regional soils (see (12). Although many local and regional sediments and soils contain low to moderate enrichment due to aeolian input (12, 13), based on comparisons to the EF standards for soils (2), several heavy metals are significantly to very highly enriched in the on- and off-site sediments as compared to local bedrock and regional soils. For example (Fig. S5), Cu is significantly enriched along on-site profiles BN and NT, while only moderately enriched along profile WD, and off-site profile P2. The most notable example is Pb (Fig. S6), which ranges from moderately to significantly enriched along off-site profile P3 and moderately enriched along P4, and on-site Profile WD, but ranges from very high to extremely high enrichment along on-site Profiles NT and BN.

**
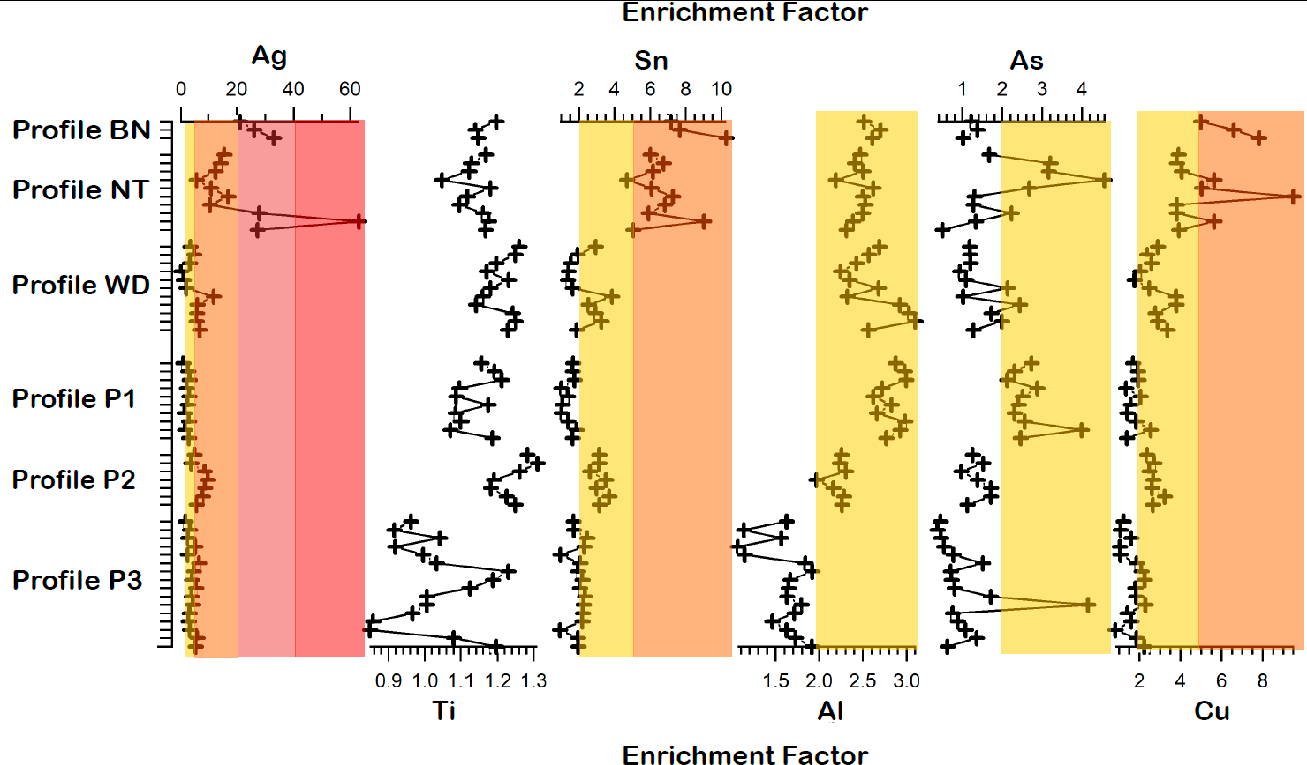
**

**Fig. S6.** Enrichment Factor (EF) along all profiles for several heavy metals. Highlighted colors represent the degree of enrichment (according to (55)) as follows: no color, EF <2, deficiency to minimal enrichment; yellow, EF 2-5, moderate enrichment; orange, EF 5-20, significant enrichment; dark pink, EF 20-40, very high enrichment; dark red, EF >40, extremely high enrichment.

**
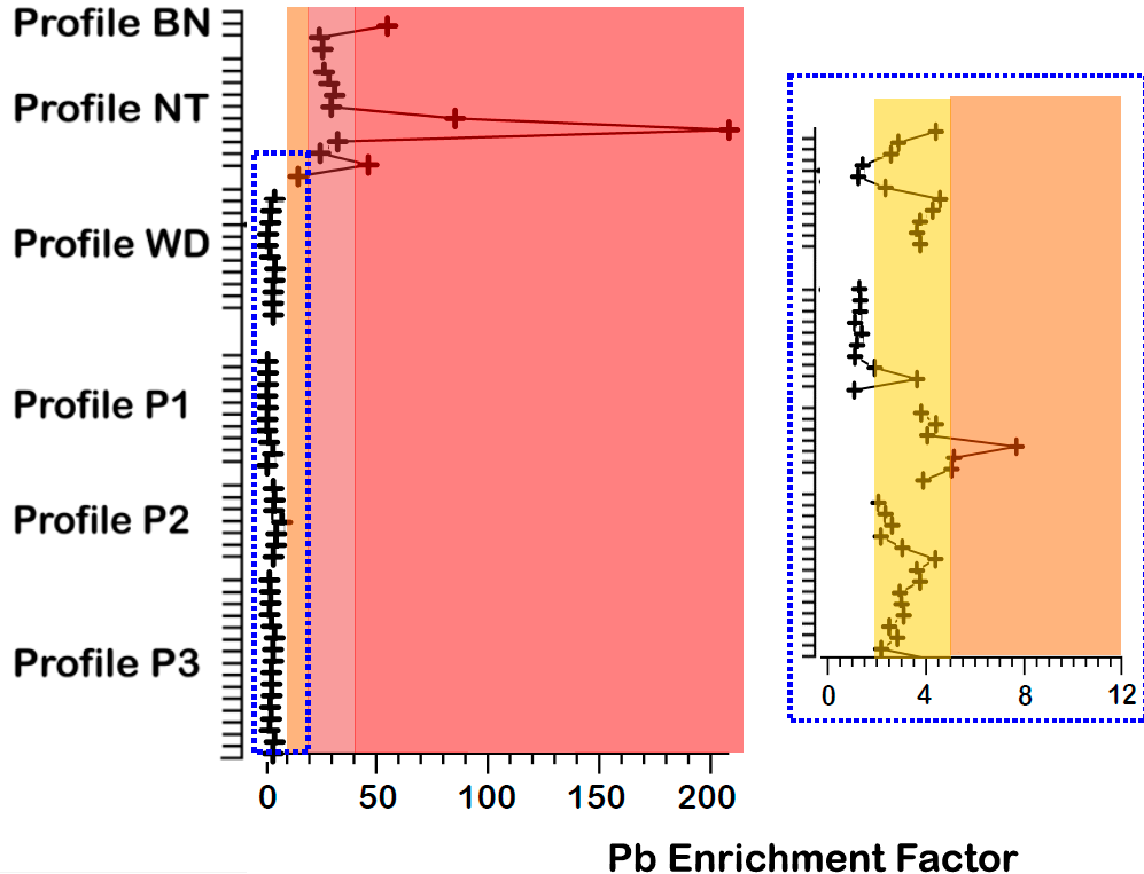
**

**Fig. S7.** Enrichment Factor (EF) along all profiles for Pb. The Pb heavy metal values along profiles highlighted by a dark blue dotted line on the main graph on the left are shown close-up in the graph delineated by a dark blue dotted line on the right. Highlighted colors represent the degree of enrichment (according to (55)) as follows: no color, EF <2, deficiency to minimal enrichment; yellow, EF 2-5, moderate enrichment; orange, EF 5-20, significant enrichment; dark pink, EF 20-40, very high enrichment; dark red, EF >40, extremely high enrichment.

Micromorphological analyses indicate that soils within the city walls contained subrounded, fine, and in many cases burned, black particulate matter, known as black carbon. There was also evidence of on-site gardening/agriculture was found in Jerash(13). The fragments of the original Red Mediterranean surface soils found beneath monuments and walls only have Pb values ranging from 9-27 ppm and Cu levels of 31-55 ppm and these values typically increase with their use as construction fill (Fig. S1, Table S2). The sewage water infrastructure sediments found on-site (Fig. S1, Dataset S1) have higher values of between 14-72 ppm for Pb and 69-115 ppm for Cu and indicates concentration in contaminants as urban sediments become waterborne within the urban area.

In summary, the on-site sediments indicate that the lowest values of Cu, Pb, Sn and Ag were located along Profile WD, the domestic outdoor space, with highest contaminant levels at or before the Early Roman period data (Figs. S2, S6 and S7, and Dataset S1). Heavy metal values of Ag, but especially Pb were wide-ranging in the Late Roman material along Profile NT, while Cu and Sn exhibited a smaller range in values. For example, values of Pb ranged from 100 to 853 ppm (median 152 ppm), Cu from 15 to 45 ppm (median 23 ppm), Ag from 47 to 1006 ppb (median 336 ppb), while Sn values varied less from 1 to 5 ppm. However, some of these fluctuating values along Profile NT belonged to culturally moved and mixed sediments (to construct later agricultural terraces), which contained much evidence for Roman aged material. The highest values from individual layers of such sediments were observed along the NT profile, including high values of Pb, in particular 350 ppm (context [8]) and 853 ppm (context [10]) along the NT Profile that represent the highest individual values obtained at Jerash (14).

Examining the spread of Pb values and EFs from Jerash as compared to Pb values obtained from other archaeological and urban settings (Figs. S8 and S9), it is observed that most EFs are below 50 with Pb values of below 200 ppm.

**
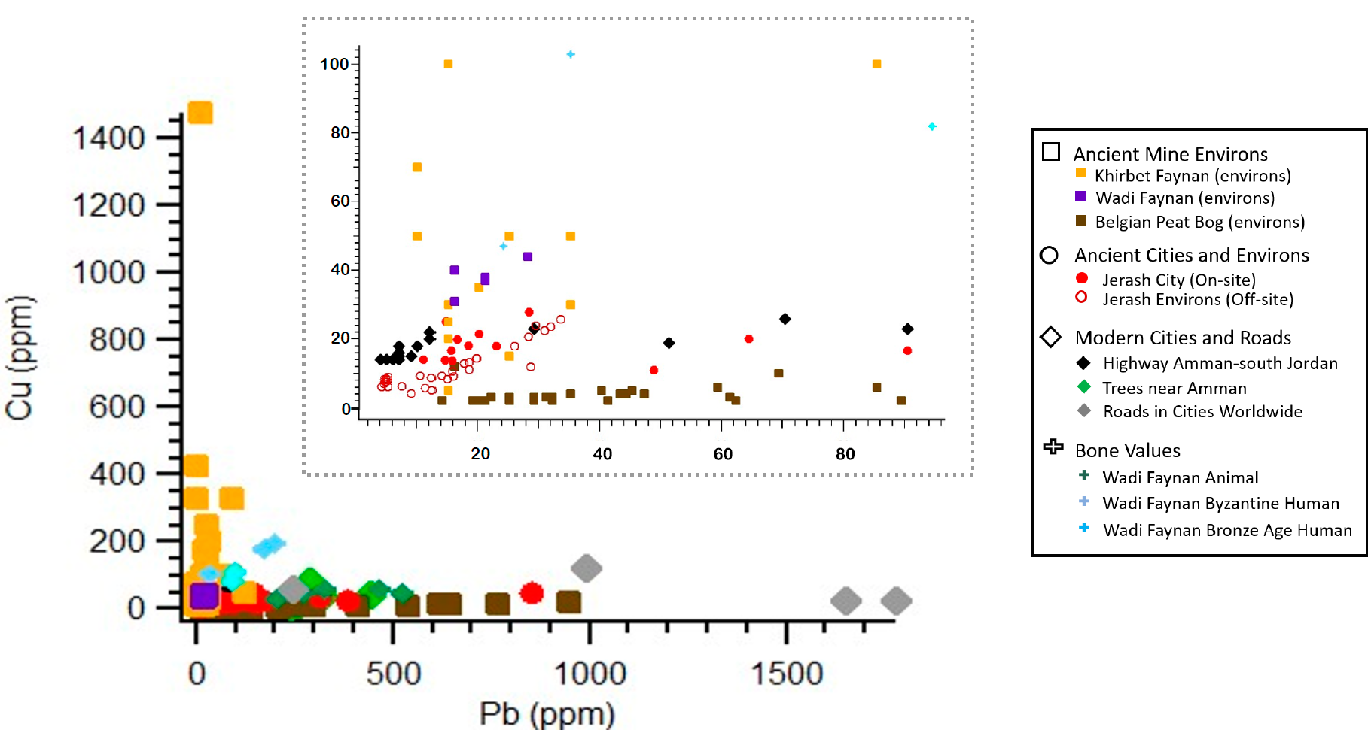
**

**Fig. S8.** Comparison of Cu and Pb values from Jerash center and environs (this study) and values from ancient mine sediments and their environs (Khirbet Faynan (6, 24); Wadi Faynan (25); (Belgian peat bog (26), modern cities and roads (Amman highway-south sediments (27); trees near Amman (28); worldwide city roads (29) and bones from near Wadi Faynan mine (9).

**
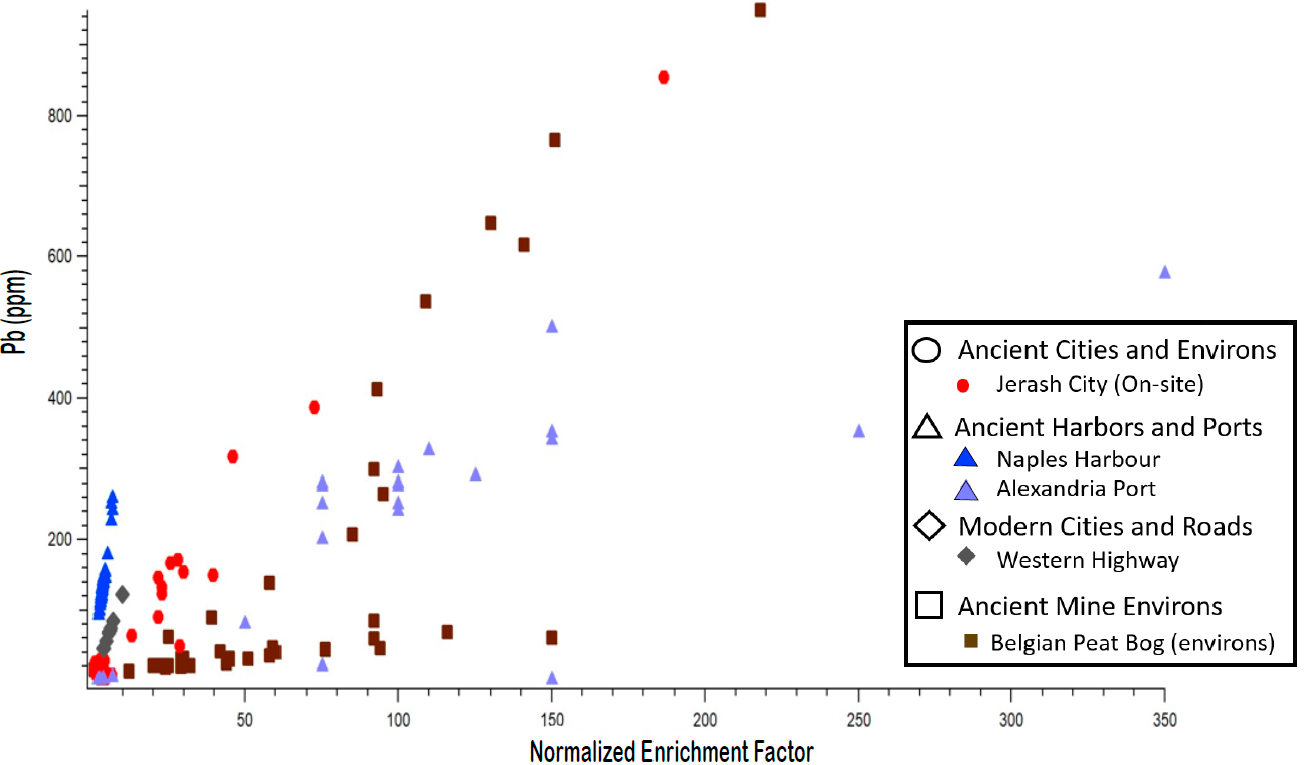
**

**Fig. S9.** Comparison of Pb and normalized EF values from Jerash center and environs and values from this study, modern cities and roads (Western Jordanian Highway (30), an ancient mine environs (26), and ancient harbors outside Roman cities (Rome’ harbor (31); Alexandria harbor/port (32)).

In comparison to Pb and Cu heavy metal values from other ancient and modern contexts (Fig. S8), the majority of Pb values at Jerash were similar to values from many peat bogs, those from the coast of Naples, and those along the modern highway in Jordan. The Pb EFs at Jerash were lower than many peat bogs EFs, but higher than those from the Neapolitan coast and the modern highway (Fig. S9). For both Cu and Pb (Fig. S8), sediments in Jerash center generally have lower values as compared to those for sediments and bones in the environs of Cu and Pb mining sites. Conversely, the values of Pb and Cu in Jerash center sediments falls within the range of values for modern urban cities and roads, though Cu in Jerash is systematically lower than that for Pb. Notably, the 853 ppm constitutes the highest Pb value obtained directly from a Roman site that was not associated with a mine in Jordan (compare data in (9, 14, 15); Figs. S8 and S9).

**Soils and sedimentology**

In the following subchapters important sedimentary and chronological on-site observations are discussed, while descriptions of profiles in the wadi are found in (12) and in (16).

**Stratigraphy.** On-site stratigraphic studies were focused on the Northwest Quarter (NWQ) of Jerash, which is located on a natural hill rising 40 m above the rest of the site. Examination of on-site urban stratigraphy allowed identification of three profiles to describe and sample in more depth, listed as follows: 1) the Basilica North Profile (BN) [372 cm depth, with 6 stratigraphic units], a continuous communal public space with the lower strata dating to the Roman to Umayyad period; 2) Trench W Profile [277 cm depth, with 10 stratigraphic units], an outdoor space, the lower strata of which have been dated to the Roman through Umayyad periods; and 3) the North Theatre Profile (NT), [455 cm depth, with 19 stratigraphic units], which was also an outdoor space. The lower strata of NT were originally associated with a street level during the Roman period, but subsequent strata consist of reused Roman-Byzantine material to create terracing used in the Ottoman-Circassian periods (Fig. 1), see (12). See also (17) and (18) about local geology.

After a survey in the Wadi Suf, we identified, described and sampled three profiles in the upper, middle and lower section of this wadi, the results of which have been summarized (12, 13, 16, 19). All soils and sediment descriptions were made using the USDA soil survey staff field book (20) and classified according to the WRB(21) system. Archaeological excavations in the NWQ at Jerash also revealed a set of buried and intact Red Mediterranean soils with typical A-horizon red (5YR4/4), and reddish brown (7.5YR 5/4) silty clay loams to clay loams overlying yellowish silty clays associated with the early city. Some of the Red Mediterranean soil samples were interpreted as being moved from outside sources into the city. Samples of buried and intact A-horizons came from Trenches O, R and Q, while transported Red Mediterranean soils came from Trenches R and W, see (13) for details.

**Descriptions of Basilica North Profile.** The BN profile has its lowest stratum/context [6] consisting of a silty clay loam with many cultural inclusions, which is overlain by a stratum [5] that is dated to the late Roman period. Context [5] contains much ash and charcoal pieces, as well as organic material in the form of black particulate matter (based on micromorphological analyses), which contributes to its very dark grey color. The overlying layers, contexts [3] and [4], probably belong to the Byzantine period as they contain some Byzantine pottery, and the layer above them (context [2]) was radiocarbon dated to the Umayyad period. A slightly lighter color (10YR 7/3 vs. 10YR 6/4), weaker structure, and more angular small pebble limestone inclusions distinguish context [3] from context [4] below. Stratum [4] has few finds, while [3] had more finds and appears to have been cultural buildup with some colluvium additions. Context [2] is a 4 cm thick, massive, ashy layer, which contains a silty sublayer with many charcoal fragments in –situ. The layer resembles a hearth-like deposit due to ash-charcoal layer as well as it appears to pinch out about 1 m to the east (see pix). The charcoal was radiocarbon dated to Late Byzantine–Umayyad period between late 6th to early 8th centuries CE (22). Overlaying the ashy layer [2], is a layer of dense cultural debris, which has been affected by colluvial processes. In the bottom 60 cm, over the surface on which a hearth or burnt material was found, are various sized boulders (e.g., 50-74 cm) in a matrix of (silty) clay loam and smaller boulders of 35-50 cm diameter. The larger boulders and mixed fill with smaller boulders, finer material and cultural debris were probably associated with a terrace wall, which was subsequently disturbed. The top 35 cm contains matrix-dominated material with gravel to small pebbles (2-4 cm diameter) in a (silty) clay loam matrix. The depth of 35 cm from the modern ground surface appears to be a surface onto which some wall-sized boulders fell in the past, possibly after the wall was abandoned.

The BN Profile offers information concerning history of the site, and site formation processes in an open, seemingly communal space at the site, which is in a relatively undisturbed area. Based on ages, the profile indicates use of space from Roman period through the Umayyad period, and after a possible hiatus in the occupation of much of the site, the use of space from the Middle Islamic through Circassian periods as well. The three lower contexts along the BN Profile context [6] have many finds associated with an open space within a cultural building. The micromorphology analysis of context [5] shows that it does have high soil organic matter content relative to all other soils, and is related to open space with a buildup up of organic debris, which is similar to other open, typically market, spaces or gardens of “anthropogenic Dark Earths” as seen in other Roman to Medieval sites in Europe (23). The various cultural, organic and burned materials also support a marketplace setting starting in the Roman/Late Roman period and lasting through the Byzantine. The overlying layer [4] contains lower amounts of heavy metals except for Pb, which is more than twice what it was before the Late Roman period. Contents of organic matter and rise of namely Pb at the end of Late Roman and into Byzantine period transition possibly signal that this area was near to areas more exclusive of metal related artisanal and/industrial activity and that organic products likely were worked here.

**Descriptions of the North Trench Profile.** The lowest layer (context [19]) consists of an AC horizon over limestone bedrock, the upper part of which is clearly disturbed, potentially by fluvial processes. Above this layer is about a meter of matrix-supported, pebble-sized clasts in a silty to silty clay loam. The lower layer of the matrix-supported deposits is context [18], which contains many cultural inclusions of pottery, glass, and mortar of Roman and possible Byzantine age. There are rip-up clasts of an Ab horizon once covering the AC horizon below, supporting water disturbances in both contexts [18] and [19], possibly associated with flooding along the road. These deposits have a late Roman age according to an OSL and several radiocarbon ages (Fig. 2; (12, 13, 16, 19)) which suggest that cultural debris built up on and near the street was impacted by a flooding in the street possibly during the late Roman to Byzantine periods. The other two layers of matrix-supported clasts, contexts [17] and [16] are fining upwards from context [18], containing fine to medium pebble and granule-sized limestone clasts and mortar fragments, respectively. Radiocarbon ages presented in (22) offered a Late Roman age (latter half of 3rd century to 4th century CE) of context [17] below, while context [16] had an earlier Roman age corresponding to the 2nd and early half of the 3rd century CE. These inverted ages suggest movement and possibly mixing of materials from around the site whereby in this case, the later sediments were eroded and deposited first, followed by the earlier deposits.

The strata above at first appeared to be similar to flash flood deposits, but several factors distinguish them as terrace deposits including clast-supported deposits with overlying mixed and randomly sorted clasts and matrix that incorporated artifacts, and a layer of relatively fine sediments covering the mixed layer, see details in (12). These sets of deposits involving pebble and cobble-sized, clast-dominated matrices (e.g., contexts [15], [12], [9], [7], and [4]), overlay mixed clasts (e.g., typically pebble-sized) and silty clay loam matrix material observed in contexts [14], [11] and [8]. Context [6] is a clast-supported matrix, similar to context [7], though with pebble-sized clasts (2-6 cm), and much pottery. Similarly, context [4], is a clast-supported layer, but with many pottery sherds, occasional mortar fragments and soil with 5YR 4/4 color. Layers [10] and [3] comprise the finer material ranging from silty sandy clay loam to silty clay loam (mentioned above) that was visible above the mixed contexts [11] and [4], respectively. Context [3] has much recent bioturbation. The upper two strata (contexts [1] and [2]) contain clearer colluvial additions, which include both sediment additions along terraces for later agriculture, as well as post-abandonment where the colluvium also includes wall tumble. Cultural inclusions are evident such as some pottery and a piece of slag. Context [1] comprises a modern soil (weak A horizon) developed in the upper part of the colluvial deposits and implies sediment stability sometime after abandonment of the terraces in this area of the site.

Contexts [5] and [13] consist of light colored and calcium carbonate rich, compacted layers. Both also contained few fine gravel limestone clasts, and many mortar inclusions, which point to their use as working surfaces, possibly in relation to terrace building. The base of boulders was observed in contexts [12], [9], [4] and [2], which infer that these layers were associated with the base and build-up of subsequent terrace walls. For example, two large boulders are stacked one on top of the other from context [12] to context [6], while other boulders are stacked from context [4] to context [1]. These possible working surfaces, contexts [13] and [5], served to create new terraces and/or add another layer to older ones.

**SI References**

1. K. H. Esbensen, A. Guyot, F. Westad, L. P. Houmøller, *Multivariate Data Analysis: In Practice : an Introduction to Multivariate Data Analysis and Experimental Design* (Aalborg Universitet, ed. 5, 2002).

2. M. Barbieri, The Importance of Enrichment Factor (EF) and Geoaccumulation Index (Igeo) to Evaluate the Soil Contamination. *J Geol Geoph* **2016** (2016).

3. F. Sulas, S. M. Kristiansen, S. Wynne-Jones, Soil geochemistry, phytoliths and artefacts from an early Swahili daub house, Unguja Ukuu, Zanzibar. *J Arch Sci* **103**, 32-45 (2019).

4. G. Holdridge, S. M. Kristiansen, A. Lichtenberger, R. Raja, I. A. Simpson, "Soils, Sediments, and urban history of the ancient City Jerash" in Environmental Studies, Remote Sensing, and Modelling*,* R. Raja, A. Lichtenberger, Eds. (Brepol, Turnhout, Belgium, 2020), vol. Jerash Paper 6, chap. 3, pp. 65-78.

5. I. A. Abboud, Geochemistry and quality of groundwater of the Yarmouk basin aquifer, north Jordan. *Environ Geochem Health* **40**, 1405-1435 (2018).

6. K. A. Knabb *et al.*, Environmental impacts of ancient copper mining and metallurgy: Multi-proxy investigation of human-landscape dynamics in the Faynan valley, southern Jordan. *J Arch Sci* **74**, 85-101 (2016).

7. L. López-Merino *et al.*, Reconstructing the impact of human activities in a NW Iberian Roman mining landscape for the last 2500 years. *J Arch Sci* **50**, 208-218 (2014).

8. A. Horváth, R. Szita, A. Bidló, Z. Gribovszki, Changes in soil and sediment properties due the impact of the urban environment. *Environ Earth Sci* **75**, 1211 (2016).

9. F. B. Pyatt, G. Gilmore, J. P. Grattan, C. O. Hunt, S. McLaren, An Imperial Legacy? An Exploration of the Environmental Impact of Ancient Metal Mining and Smelting in Southern Jordan. *J Arch Sci* **27**, 771-778 (2000).

10. A. L. Collins *et al.*, Sediment source fingerprinting as an aid to catchment management: A review of the current state of knowledge and a methodological decision-tree for end-users. *J Environ Man* **194**, 86-108 (2017).

11. A. L. Collins, D. E. Walling, G. J. L. Leeks, Source type ascription for fluvial suspended sediment based on a quantitative composite fingerprinting technique. *Catena* **29**, 1-27 (1997).

12. G. Holdridge, City and Wadi: long-term human impacts on eastern Mediterranean sediment dynamics at a catchment scale. *Geomorphology* (submitted).

13. G. Holdridge *et al.*, Urban gardens and the use of Red Mediterranean Soil in Antiquity. The case of Gerasa/Jerash in Jordan. *Geoderma* (submitted).

14. N. Abderahman, Y. H. Abu-Rukah, An assessment study of heavy metal distribution within soil in upper course of Zarqa River basin/Jordan. *Environ Geol* **49**, 1116-1124 (2006).

15. B. Lucke, H. Kemnitz, R. Bäumler, M. Schmidt, Red mediterranean soils in jordan: New insights in their origin, genesis, and role as environmental archives. *Catena* **112**, 4-24 (2014).

16. A. Lichtenberger, R. Raja, E. H. Seland, T. Kinnaird, I. A. Simpson, Urban-Riverine Hinterland Synergies in Semi-Arid Environments: Millennial-Scale Change, Adaptations, and Environmental Responses at Gerasa/Jerash. *J Field Arch* **44**, 333-351 (2019).

17. G. Holdridge, "The Geology of the Northwest Quarter of Ancient Jerash within its Regional Context" in Environmental Studies, Remote Sensing, and Modelling: Final Publications from the Danish-German Jerash Northwest Quarter Project, Jerash Papers 6*,* A. Lichtenberger, R. Raja, Eds. (Brepols, Turnhout, 2020).

18. N. Abu-Jaber, "The Quarryscapes of Gerasa (Jarash), Jordan" in Geological Survey of Norway Special publications no. 12. (Oslo, 2009), pp. 65-73.

19. A. Cresswell *et al.* (2018) Luminescence dating of soils and sediments from Jerash, Jordan. in *Biological and Environmental Sciences Research Reports* (Sterling University, Scotland), p http://hdl.handle.net/1893/27524.

20. S. Soil Survey, *Keys to soil taxonomy* (United States Department of Agriculture, Washington, 2003), vol. 9th edition, pp. 1-332.

21. I. W. G. WRB (2006) World Reference Base for Soil Resources 2006. (Rome), pp 1-128.

22. B. Philippsen, J. Olsen, "Dating the undatable: pretreatment and radiocarbon dating of human bones with extremely low collagen preservation from Jerash" in Final Publications from the Danish-German Jerash Northwest Quarter Project, Jerash Papers, 6*,* A. Lichtenberger, R. Raja, Eds. (Brepols, Tourhout, 2020), pp. 139-156.

23. B. Wouters, C. Makarona, K. Nys, P. Claeys, Characterization of Archaeological Metal Remains in Micromorphological Thin Sections Using μXRF Elemental Mapping. *Geoarchaeology* **32**, 311-318 (2017).

24. J. P. Grattan, D. D. Gilbertson, M. Kent, Sedimentary metal-pollution signatures adjacent to the ancient centre of copper metallurgy at Khirbet Faynan in the desert of southern Jordan. *J Arch Sci* **40**, 3834-3853 (2013).

25. M. A. Beherec *et al.*, Iron Age Nomads and their relation to copper smelting in Faynan (Jordan): Trace metal and Pb and Sr isotopic measurements from the Wadi Fidan 40 cemetery. *J Arch Sci* **65**, 70-83 (2016).

26. F. De Vleeschouwer *et al.*, Atmospheric lead and heavy metal pollution records from a Belgian peat bog spanning the last two millenia: Human impact on a regional to global scale. *Sci Total Environ* **377**, 282-295 (2007).

27. F. Howari, Y. Nazzal, P. Goodell, Heavy metal pollution of soils along North Shuna-– Aqaba Highway, Jordan. *Int. J Environ Polut* **22**, 597-607 (2004).

28. H. El-Hasana, H. Al-Omaria, A. Jiriesb, F. Al-Nasirc, Cypress tree (Cupressus semervirens L.) bark as an indicator for heavy metal pollution in the atmosphere of Amman City, Jordan. *Environ inter* **28**, 513–519 (2002).

29. J. M. Trujillo-Gonzalez, M. A. Torres-Mora, S. Keesstra, E. C. Brevik, R. Jimenez-Ballesta, Heavy metal accumulation related to population density in road dust samples taken from urban sites under different land uses. *Sci Total Environ* **553**, 636-642 (2016).

30. Q. M. Jaradat, A. M. Massadeh, K. A. Momani, M. A. Al Saleem, The Spatial Distribution of Pb, Cd, Zn, and Cu in Agricultural Roadside Soils. *Soil Sedi Contam* **19**, 58-71 (2010).

31. H. Delile *et al.*, Rome’s urban history inferred from Pb-contaminated waters trapped in its ancient harbor basins. *Proc Nat Ac Sci* **114**, 10059-10064 (2017).

32. A. Véron, J. P. Goiran, C. Morhange, N. Marriner, J. Y. Empereur, Pollutant lead reveals the pre-Hellenistic occupation and ancient growth of Alexandria, Egypt. *Geophys Res Lett* **33** (2006).

**Table S1 (separate file).** Statistical analyses on the heavy metal values using Welch’s ANOVA. See Dataset S1 for abbreviations

**Table S2 (separate file).** Statistical analyses on the heavy metal values using post hoc Games Howell tests due to unequal variances. See Dataset S1 for abbreviations

**Dataset S1 (separate file).** Measured elemental concentration from Jerash and its hinterland.
