# Supplementary material for: A Roman provincial city and its contamination legacy from artisanal and daily-life activities
Source: PLoS One. 2021 Jun 9;16(6):e0251923. doi: 10.1371/journal.pone.0251923 (PMC8189455; doi:10.1371/journal.pone.0251923)
Supplement: Attachment — Submitted filename: Response to reviewers.docx [file pone.0251923.s005.docx]

Authors’ respond to reviewer comments

*Journal ID: PONE-D-21-01035*

*Title: “A Roman provincial city and its contamination legacy from artisanal and daily-life activities”*

*Authors: Genevieve Holdridge, Søren M Kristiansen, Gry H Barfod, Tim C. Kinnaird, Achim Lichtenberger, Jesper Olsen, Bente Philippsen, Rubina Raja, Ian Simpson*

The authors greatly appreciate the reviewers’ time and expertise, which resulted in valuable comments. Thank you! For easy reference, we have numbered each specific comment below, while our answers are highlighted in blue.

**Comments from Reviewer: 1**

**General comments**

C0.1: Overall: This is a well prepared manuscript which has explored the social-technological-environmental relationships in the ancient city of Jerash. The data presented are interesting but there are questions in the way the results are interpreted. A major concern is with the sources of metals to account for the large-scale contamination of Gerasa/Jerash and its hinterlands. The level of heavy metal loading into the environment is clearly out of balance with what would be expected from “small-scale but common Roman urban, artisanal and everyday site activities”. The authors claim that the main uses of metals in the city included artisanal metalworking activity, production of copper coinage and making of pigments. The environmental footprints of such activities in all likelihood were highly localized within the residential areas. The total number of people involved in such activities was probably small considering that Jerash was a famous trading center and was not particularly known for its meta-working prowess. The question of where did all the metal pollution come from was not answered satisfactorily in the paper. The authors should consider the possibility that there was a lost lead/gold or lead/silver mine in or near the city which had released enough metalliferous wastes and fumes to contaminate the entire city and its neighborhood..

Answer: A lost mine close to Jerash would not explain the higher metal contamination in the city compared to the hinterland wadi and down-stream only, unless the mine was in the city itself. In this case, there should have been clear signs of such a mine or at least the activities surrounding it and suitable geology, which is not the case (see references in the article). Slags from the intense mining at the Cu mines about 300-400 km to the South in Feinan and Timnah show high concentrations of Cu, Pb, Zn, Cd and As, but neither airborne pollution from here cannot explain the higher metal levels in Geresa compared to the wadi hinterland. Transportation of this pollution into Geresa through people migration or import of polluted agricultural goods from south would be purely speculative and not supported by the fact that Geresa hinterlands are more fertile than further south. Instead, the elevated copper, lead and silver concentrations in Jerash’ urban sediments are present in copper and lead artefacts and given that silver is less soluble than copper and lead, it will tend to accumulate in the soils despite its lower overall presence in the artefacts. While attracted to the idea that there was a mine in or near the city there is no evidence to support this hypothesis. To give a more nuanced assessment of our data we highlight the localised but intense urban activity associated with metals in the city and we clarify that our basis for considering hinterland are the sediments of wadi suf only by including the term “wadi hinterland” in the appropriate cases. We hope this answer also answers some of C1.4, C1.6, C1.7 and C1.9.

**Specific Comment:**

*C1.1 Comment 1 (Lines 119-123): The documentation of the source-sink relationships between the concentrations of metals in urban soils and Wadi Suf sediments is weak. The following statements (lines 119-123) support this concern: “Evidence of fluvial sedimentation from the 2nd-3rd centuries BC to the 7th century CE remained elusive, suggesting effective management of the water and soil resources at this time. From the beginning of the 6th century, the wadi begins to in-fill, and in the absence of additional chronological data may imply a decline in land and water management with associated erosion of soils to the wadi”. It should be noted that the graphs (of Cu and Pb concentrations at the specific locations) in Figure 3 do not include the time information for the samples. The peaks in the graph should be interpreted with caution unless the samples are not of the same age.*

Answer: We have elaborated our descriptions to address this concern about the functioning of the wadi as a sediment trap as contamination and erosion/sedimentation are different processes. There can be long periods when the sediments in the city are contaminated, but not eroded down into the wadi, due to a functioning management of the soil and cityscape. During periods with less efficient soil management, the (contaminated) urban soils can be eroded into the wadi. This lead to a time lag between contamination of soil in the city and sedimentation of contaminated sediments in the wadi downstream. In light of comment C1.1, we have clarified the text in lines 119-123 to make clear the contaminated wadi sediments are later in their deposition than the contamination of sediments in the urban area, and, in doing so also addresses the Figure 3 comment made by the reviewer.

*C1.2 Comment 2: The role of local climate conditions has not been adequately addressed. There are reports suggesting that the late Roman Empire period was marked by major climatic changes in the Middle East which could have had some influence in the supply and accumulation of sediments in the river basin. A major floor can easily mediate the amount and type of sediments deposited at any location in the Wadi Sufa basin. Interpreting the sedimentary record solely on the basis of human activities (without consideration of the geophysical and geochemical processes) is an over-simplification.*

Answer: We agree that the human—influenced and polluted sediments from the city only later could accumulated in the wadi sediments, likely after being eroded and redeposited by flash floods and similar extreme climate events. We have hence included recognition of the role of climate change and earthquake, as well as humans in sediment movement (line 123-).

*C1.3 Comment 3 (Figure 2): What is the error range in the stimulated results?*

Answer: This has been clarified in the materials and methods section by describing that the 14C errors for the simulated 14C dates are drawn from a probability distribution which is constructed from the actual 14C data, and hence that the errors resemble the errors of the actual measurements.

*C1.4 Comment 4: The following statement (lines 138-141) cannot be justified: “We then measure and compare heavy metal concentrations in two sets of chronologically controlled stratigraphies, from within the city and from the Wadi Suf hinterland (13-15). The spatial and temporal relationships between these stratigraphic units enable assessment of local pathways, from sources to sinks”. It would be an impossible task (almost) to reliably infer the transfer pathways for the metals in the particular environment based on their concentrations in such divergent locations. In the first instance, lead is immobile in soils and the mechanism for its transfer from within the city to the Wadi Suf hinterland needs to be explained..*

Answer: We have changed the emphasis of this statement to give the assessment as an indication. We agree that lead, for example, is largely fixed in soil; however, it is the whole sediment movement, with lead as part of the transported particles that concerns us, and which ends up in the wadi hinterland downstream. We have done our best to clarify this in our text in several places with minor edits including “wadi hinterland” a.o. Please also see C0.1.

*C1.5: Comment 5 (Figures 5, S6 and S7): It is difficult to infer any historical relationships in these figures without showing any time lines. These figures probably should be redone.*

Answer: We agree that the chronology is not shown in these figures. However, the purpose of supplementary information figures S5-S7 are to highlight the smaller elemental differences in the on-site vs. the off-site pollution and how they are distributed along the course of the wadi. Nearly all of them are non-modern strata and hence must contain pre-modern contamination. We prefer accordingly to keep the present version as we feel that inclusion of more explanatory labels/axes (e..g time) in the already complicated figures would reduce its readability significantly. Please see Figure 2 for the chronology of the individual sites.

*C1.6: Comment 6 (lines 221-222): What is the evidence that the weathering of metal-based materials and artefacts represents “significant secondary sources” of environmental metal contamination in the city? Even if so, the impacts would be very highly localized.*

Answer: *As far as we understand this comment, the reviewer wants to know specifically what the evidence of the contribution of weathering is, compared to e.g. the production and use of those materials and artefacts. This statement has now been qualified in the text. Please see answer C0.1 as well*

*C1.7: Comment 7 (lines 244-248): The statement that elevated silver concentrations of Jerash sediments came from secondary sources such as Pb-rich material since some galena ores contain significant amounts of silver (argentiferous galena) is interesting. It hints at a likelihood that lead ores might have been exploited in the area in ancient times. The lead deposits would have been exhausted by now..*

Answer: please see answer to C0.1. We could add to our answer here that, in the absence of local mined sources, galena likely was traded over long distances, so that lead deposits in the vicinity of the city were not a necessity. The origin of the silver contamination is not clear from our data, but we suggest that the Ag could have been introduced with the lead and contaminated the soils due to coin or object production or when the corroded objects ended up in the soil.

*C1.8: Comment 8 (Figure S5): The difference in the spread of Pb and Cu concentrations in on-site versus off-site sediments needs to be explained*

Answer: The interpretation of this spread has been described better in the text to Figure S5.

*C1.9: Comment 9 (Conclusions): The study purportedly shows that most of the lead and copper released into the environment in Jerash city are deposited locally. There is no evidence to suggest that the metal works in the city were able to release much of the metals into the atmosphere for regional and global circulation. The conclusion that “small-scale, daily-life use and reuse activities in urban settings are an important factor in understanding and explaining changes in the baseline of global pollution, not only today, but over the last millennia” is misleading.*

Answer: We strongly believe that our data show that the anthropogenic activities in the ancient city of Jerash is a significant local source of pollution of the urban soils and the wadi sediment. However, in respect to the “...*no evidence to suggest that the metal works in the city were able to release much of the metals into the atmosphere for regional and global circulation*” we agree that our data tells little about the “regional and global” scales. On the other hand, all published regional and global studies of ancient air pollution only consider (see refs in paper) the first production at the mines as a source. This is, however, not what contemporary air pollution studies suggest, namely that many small point sources also have a significant effect on increasing pollution baselines in the ambient environment. This paragraph in the conclusion has hence been added the word “potential”. This, we hope, reflects better that our data show long-lasting local pollution from the ancient city, including artisanal metal working activities, which we believe warrants more studies of multi-source urban use and reuse of metals as a potential source of pollution in future regional and global palaeo-pollution studies. Please see answer to C0.1 as well.

**Comments from Reviewer: 2**

*General Comments:*

*The data, methodologies, and conclusions are all clearly presented and discussed. The findings are significant for the archaeological community and for other urban studies disciplines. I endorse publication.*

Answer: Thank you for the nice comment!
